# Medical education videos – comparative analysis of sonography vs. clinical examination videos: user perception and educational value

**DOI:** 10.1186/s12909-024-06478-9

**Published:** 2024-12-18

**Authors:** Timur H. Kuru, Johannes Salem, Emrah Hircin, Carolin Siech, Nadim Moharam, Justine Schoch, Angelika Mattigk, Julian Struck, Hendrik Borgmann, Tim Nestler

**Affiliations:** 1 Department of Urology, Federal Armed Forces Hospital Koblenz, Koblenz, Germany; 2 Department of Urology, University of Cologne, Cologne, Germany; 3 AMBOSS GmbH, Cologne, Germany; 4 Goethe University Frankfurt, University Hospital, Department of Urology, Frankfurt, Germany; 5 Cancer Prognostics and Health Outcomes Unit, Division of Urology, University of Montréal Health Center, Montréal, Québec Canada; 6 Department of Urology, University of Mannheim, Mannheim, Germany; 7 Department of Urology and Pediatric Urology, University of Ulm, Ulm, Germany; 8 Department of Urology, Faculty of Health Sciences Brandenburg, Brandenburg Medical School Theodor Fontane, Brandenburg an der Havel, Germany; 9 CUROS Urology Center, Klinik LINKS VOM RHEIN, Cologne, Germany

**Keywords:** Medical education, Educational video, Video quality, Medical training.

## Abstract

**Background:**

Video content has become an increasingly valuable tool in medical education, particularly for teaching hands-on skills like sonography and clinical examination. This study evaluates the satisfaction and content of sonography and clinical examination videos on the AMBOSS platform, a prominent medical education resource in Germany.

**Objective:**

The goal of this study was to compare how effective and well-received sonography and clinical examination videos are on the AMBOSS platform. The study looked at aspects such as the medical and technical quality of the videos, their usefulness for learning, and overall user satisfaction.

**Methods:**

Eighteen instructional videos were chosen and made accessible on the AMBOSS platform, grouped into sonography (*n* = 9) and clinical examination (*n* = 9) categories. Users were asked to voluntarily and anonymously fill out a questionnaire evaluating the videos. Over 49.5 months, data from 1,643,274 video views and 936 completed questionnaires were gathered.

**Results:**

Clinical examination videos were watched significantly more often than sonography videos (86 vs. 14%). Both video types were highly rated in terms of medical and technical quality. However, sonography videos were judged superior in technical quality and clarity, whereas clinical examination videos were preferred for their medical quality and practical application. Feedback from users indicated a desire for more detailed annotations and clearer explanations.

**Conclusion:**

The findings underline the crucial role of video resources in medical education, particularly in teaching practical skills. To improve educational outcomes, it is important to tailor content to the specific needs of medical students and professionals, incorporate user feedback, and take advantage of technological advancements.

**Supplementary Information:**

The online version contains supplementary material available at 10.1186/s12909-024-06478-9.

## Introduction

Medical education increasingly relies on video content to improve learning outcomes for students and professionals [1, 2]. This approach is particularly effective for teaching practical skills like sonography and clinical examinations. This is especially true for practical skills like sonography and clinical examinations, where visual demonstrations can deepen understanding and reinforce memory for complex procedures [3]. By integrating videos into structured training programs, learners gain access to a more dynamic and interactive learning experience compared to traditional methods such as textbooks and lectures [4]. This study compares the effectiveness and content of sonography and clinical examination videos offered on the AMBOSS platform, an established resource in this field in Germany.

The topic of instructional videos in medicine has been explored in various studies. For instance, Nason et al. [5] reported an improvement in training for male urinary catheterization through a related YouTube video, while Armstrong et al. [6] demonstrated enhanced psoriasis diagnostics via an instructional video on a dermatological platform. Recent technological advances in video quality, such as high-definition and 3D visualization, have also contributed to a richer learning experience. These improvements are particularly advantageous for skills like sonography, where understanding spatial anatomy and real-time imaging are crucial [7]. Videos with strong technical and medical quality can bridge the gap between theoretical knowledge and hands-on practice, providing learners with a clear, accessible pathway to clinical proficiency [8].

This study focuses on 18 medical training videos from the AMBOSS platform, divided into sonography and clinical examination categories. Viewers were invited to complete a questionnaire that assessed the medical and technical quality of the videos, their usefulness for learning, and overall satisfaction. The study’s main goal is to analyze which topic in medical education can be addressed most appropriate by video education. To address this important, unsolved question, we analyzed the data from these questionnaires to evaluate how effective the sonography videos are compared to the clinical examination videos.

## Materials and methods

### Distribution of the questionnaire

A selection of 18 medical teaching videos **(**Table 1**)** was implemented in the corresponding chapters on the teaching platform www.amboss.com in 2019. The Amboss platform is a mainly campus licensed access restricted educational website with educational content in human medicine. The videos were created by a medical editorial team with extensive experience in producing didactic teaching videos. An interprofessional team of medical professionals, videographers, and sound technicians collaborated on the production. The videos were developed following a standardized didactic concept. After production, the content was reviewed by subject matter experts to ensure accuracy and quality.

**Table 1:** Selection of 18 educational videos - categorized by clinical examination (*n* = 751 (80%)) and sonography (*n* = 185 (20%)), which were implemented on amboss.com. The frequency of video views with a completed questionnaire is given as an absolute number and relative to the total cohort n (%)

Video	Category	Views *n* (%)
Clin. ex. Abdomen	Clinical examination	50 (5)
Clin. ex. Blumberg sign	Clinical examination	30 (3)
Clin. ex. hepatojugular reflux	Clinical examination	48 (5)
Clin. ex. Heart	Clinical examination	108 (12)
Clin. ex. Lungs	Clinical examination	70 (8)
Clin. ex Ratschow storage test	Clinical examination	54 (6)
Clin. ex. Riva Rocci blood pressure measurement	Clinical examination	32 (3)
Clin. ex Thyroid gland	Clinical examination	315 (34)
Clin. ex. Central venous pressure	Clinical examination	44 (5)
Clinical examination videos		751 (80)
Sono Abdominal vessels	Ultrasound	16 (2)
Sono Image setting and mode of operation	Ultrasound	20 (2)
Sono Gallbladder	Ultrasound	17 (2)
Sono Urinary bladder	Ultrasound	4 (0)
Sono Liver	Ultrasound	40 (4)
Sono Spleen	Ultrasound	7 (1)
Sono Kidney	Ultrasound	6 (1)
Sono Pancreas	Ultrasound	6 (1)
Sono Thyroid gland	Ultrasound	69 (7)
Total sonography videos		185 (20)
**Total**		**936**

Clin. Ex. = clinical examination; Sono = Sonography

In the period from 25.03.2019 to 08.05.2023 (49.5 months), a uniform questionnaire was displayed after these videos for voluntary and anonymous responses. The number of views of the individual videos was also recorded via the amboss.com metadata. This article does not include any studies on humans or animals, which is why ethical approval was waived.

### Questionnaire

The online questionnaire for the assessment and evaluation of the educational video comprised a total of 12 items in German on medical, technical and didactic quality, usefulness for own knowledge acquisition, recommendation and socio-demographic data of the respondents (questionnaire in English translation: Supplementary material Table 1). Aspects of video quality were examined using acceptability e-scale for five of the six-questions. The content of these questions was modified for the videos to be examined here [2]. These were to be answered using a 5-point Likert scale. The sixth question covered the aspects of general satisfaction, medical quality, technical quality, didactic usefulness and time-related usefulness. Furthermore, the general video quality was recorded using the Global Quality Score translated into German, also using a 5-point Likert scale [1]. If it was stated that the time taken was inappropriate in relation to the content conveyed, the respondents were asked whether the video was perceived as too long or too short. The section ended with the free text question: “What did you feel was missing from the video? What do you think could have been left out?“.

The sociodemographic data collected included gender (female, male, diverse, not specified), level of training (student, medical doctor in specialist training, specialist) and, depending on the level of training, the question about the semester, year of specialist training or years as a specialist. The survey was concluded with the open question: “Do you have any further comments? We look forward to your feedback!“.

### Videos

The implemented videos on www.amboss.com are intended for additional teaching of medical students in order to expand and improve their practical skills. Accordingly, the video contents focus on clinical examination and sonography – the specific video contents are depicted in **Tabel 2**. Additionally, the video length was limited to few minutes and all videos had an audio comment in order to use the audiovisual effect and explain what is shown. The videos were created by a medical editorial team with extensive experience in producing didactic teaching videos. An interprofessional team of medical professionals, videographers, and sound technicians collaborated on the production. The videos were developed following a standardized didactic concept. After production, the content was reviewed by subject matter experts to ensure accuracy and quality.

### Statistical analysis

Absolute and relative frequencies were specified for categorical variables, median and range (minimum and maximum value) were specified for continuous variables. The chi-square test was used for the comparison of nominally and ordinally scaled variables, the Wilcoxon Rank-Sumtest for interval-scaled, independent samples. All statistical tests were 2-sided. A p-value < 0.05 was considered significant. The statistical calculations were performed with SPSS 29.

## Results

### General

During the survey period, the videos analyzed here and implemented on the amboss.com were viewed a total of 1,643,274 times, of which 1,412,011 (86%) were clinical examination videos and 231,263 (14%) were sonography videos **(**Table 2**)**. These were a total of 936 answered online questionnaires (Table 1) - categorized by clinical examination (*n* = 751 (80%)) and sonography (*n* = 185 (20%)).

**Table 2:** Selection of 18 educational videos - categorized by clinical examination and sonography which were implemented on amboss.com. The frequency of video views is given as absolute number and relative to the total cohort n (%). In addition, detailed information about average playback times and Youtube ratings (like / dislike) are displayed. The relative values to the likes / dislikes refer to the total number of likes / dislikes per video

Video	Views *n* (%)	Average playback time (%)	Like *n* (%)	Dislike *n* (%)
Clin. ex. Abdomen	275,961 (17)	44	2,153 (98)	52 (2)
Clin. ex. Blumberg sign	96,243 (6)	67	559 (98)	10 (2)
Clin. ex. hepatojugular reflux	74,941 (5)	60	475 (99)	7 (1)
Clin. ex. Heart	234,770 (14)	61	1,612 (97)	48 (3)
Clin. ex. Lungs	318,617 (19)	53	2,232 (98)	47 (2)
Clin. ex Ratschow storage test	56,655 (3)	76	242 (97)	8 (3)
Clin. ex. Riva Rocci blood pressure measurement	169,313 (10)	57	1,544 (98)	38 (2)
Clin. ex Thyroid gland	116,191 (7)	72	722 (98)	16 (2)
Clin. ex. Central venous pressure	69,320 (4)	66	507 (96)	20 (4)
**Clin. ex. videos**	**1**,**412**,**011 (86)**		**10**,**046 (98)**	**246 (2)**
Sono Abdominal vessels	26,801 (2)	45	227 (96)	10 (4)
Sono Image setting and mode of operation	12,224 (1)	65	156 (9)	1 (1)
Sono Gallbladder	67,034 (4)	57	548 (98)	12 (2)
Sono Urinary bladder	15,450 (1)	62	125 (100)	0 (0)
Sono Liver	33,312 (2)	51	222 (100)	3 (0)
Sono Spleen	9,753 (1)	65	81 (100)	0 (0)
Sono Kidney	26,851 (2)	62	218 (99)	2 (1)
Sono Pancreas	17,435 (1)	61	150 (100)	1 (0)
Sono Thyroid gland	22,403 (1)	60	239 (99)	3 (1)
**Total sonography videos**	**231**,**263 (14)**		**1**,**966 (98)**	**32 (2)**
**Total**	**1**,**642**,**274**		**12**,**012 (98)**	**278 (2)**

Clin. Ex. = clinical examination; Sono = Sonography

### YouTube metadata

All 18 videos were viewed 91,293 (SD 95,843) times on average. Separated according to clinical examination and sonography, nine videos each, these were viewed on average 156,890 (SD 97,672) vs. 25,696 (17,281) *p* = 0.004. In addition, some videos were rated at a low threshold using the YouTube like / dislike function. 10,046 (0.7%) of the clinical examination videos and 1,966 (0.9%) of the sonography videos were liked, 246 (0.02%) of the clinical examination videos and 32 (0.01%) of the sonography videos were disliked.

### Socio-demographic data

**Fig. 1:**
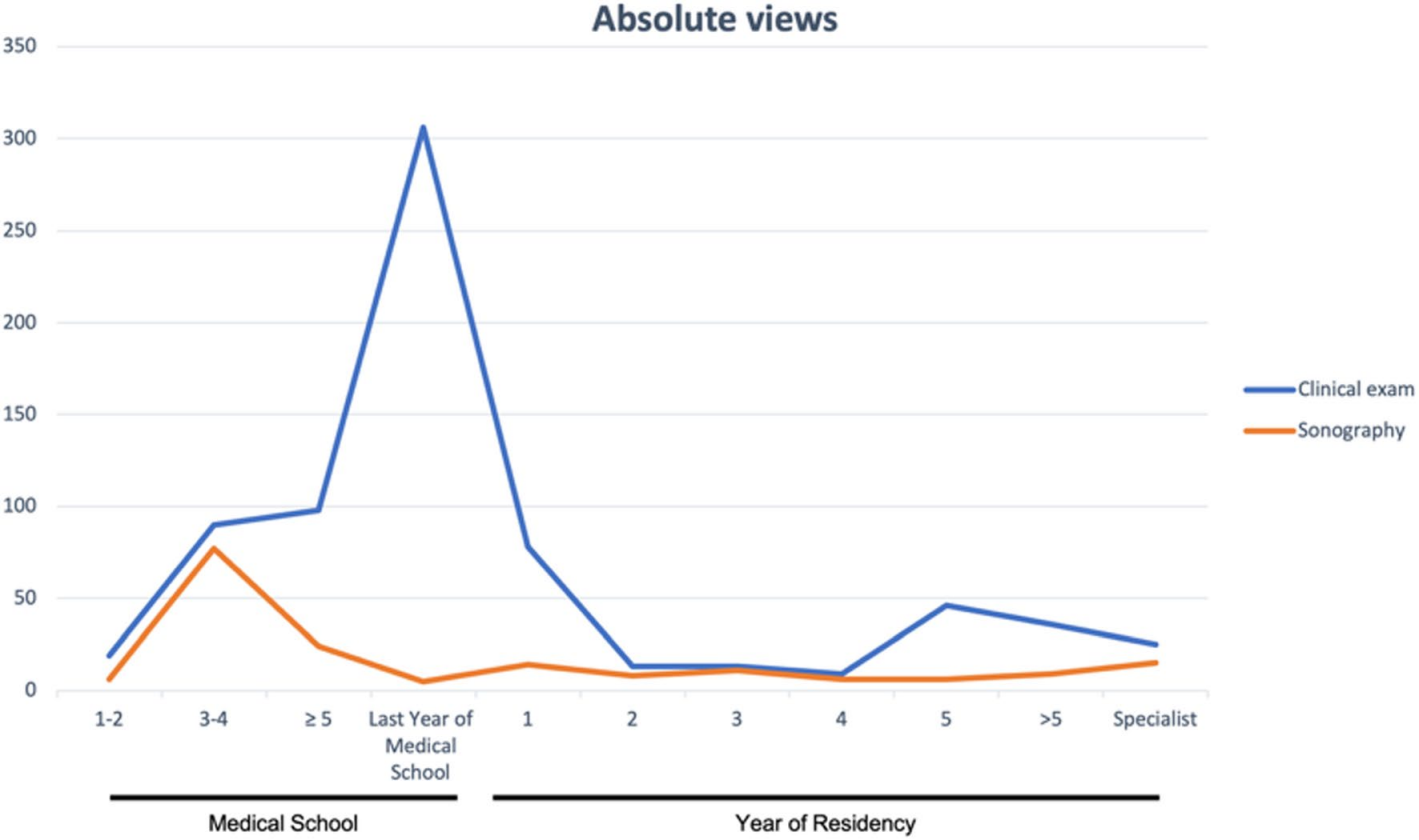
Absolute number of views per clinical examination video vs. sonography videos separated by training level

The socio-demographic data of the survey participants are shown in Table 3, separated according to clinical examination and sonography. Both gender and level of education were similarly distributed between the two groups (*p* = 0.12 female vs. male). Regarding the level of training, video users were significantly more likely to be students than doctors (*p* = 0.003) and, when comparing the year of training, most users were in the last year of medical school (< 0.001). Figure 1 shows the absolute usage of the videos. The videos are mainly watched during medical school, with a slightly earlier peak for ultrasound videos in the 3rd to 4th year of study and the clinical examination videos in the last year of medical school.

**Table 3:** Sociodemographic data of survey participants separated by clinical examination and sonography. Data are given as n (%)

Variable	Clinical exam	Sonography	*p*-value
**Sex**			0.27
Female	482 (64)	109 (59)	
Male	237 (32)	70 (38)	
diverse	6 (1)	0 (0)	
Not specified	11 (1)	3 (2)	
Missing	15 (2)	3 (2)	
**Level of training**			0.003
Student	513 (68)	112 (61)	
MD in training	198 (26)	54 (29)	
MD specialist	25 (3)	16 (9)	
missing	15 (2)	3 (2)	
**Year of training**			< 0.001
**Year of medical school**	513 (68)	112 (61)	
1–2	19 (4)	6 (5)	
3–4	90 (18)	77 (69)	
>= 5	98 (19)	24 (21)	
Last year of medical school	306 (60)	5 (4)	
**Year of residency**	195 (26)	54 (29)	
1	78 (40)	14 (26)	
2	13 (7)	8 (15)	
3	13 (7)	11 (20)	
4	9 (5)	6 (11)	
5	46 (24)	6 (11)	
> 5	36 (18)	9 (17)	
**Years since specialisation**	25 (3)	16 (9)	
**missing**		3 (2)	
**Total**	751 (80)	185 (20)	

### Video quality (questions 1–6)

The response rates to the questions on video quality were very good, as shown in Table 4. In general, both the clinical examination videos and the sonography videos were rated very well. Responses < 4 in the mean value per question were only shown for question 1 on the “first impression of the video” 4x for clinical examination and 2x for sonography. However, it should be noted that the lowest mean score was still a good 3.78. An excellent rating of exclusively 5 on the Likert scale was given for kidney sonography (questions 1–5) and urinary bladder sonography (questions 1–2). However, the relatively low number of views of 4 and 6 should be noted here.

**Table 4:**
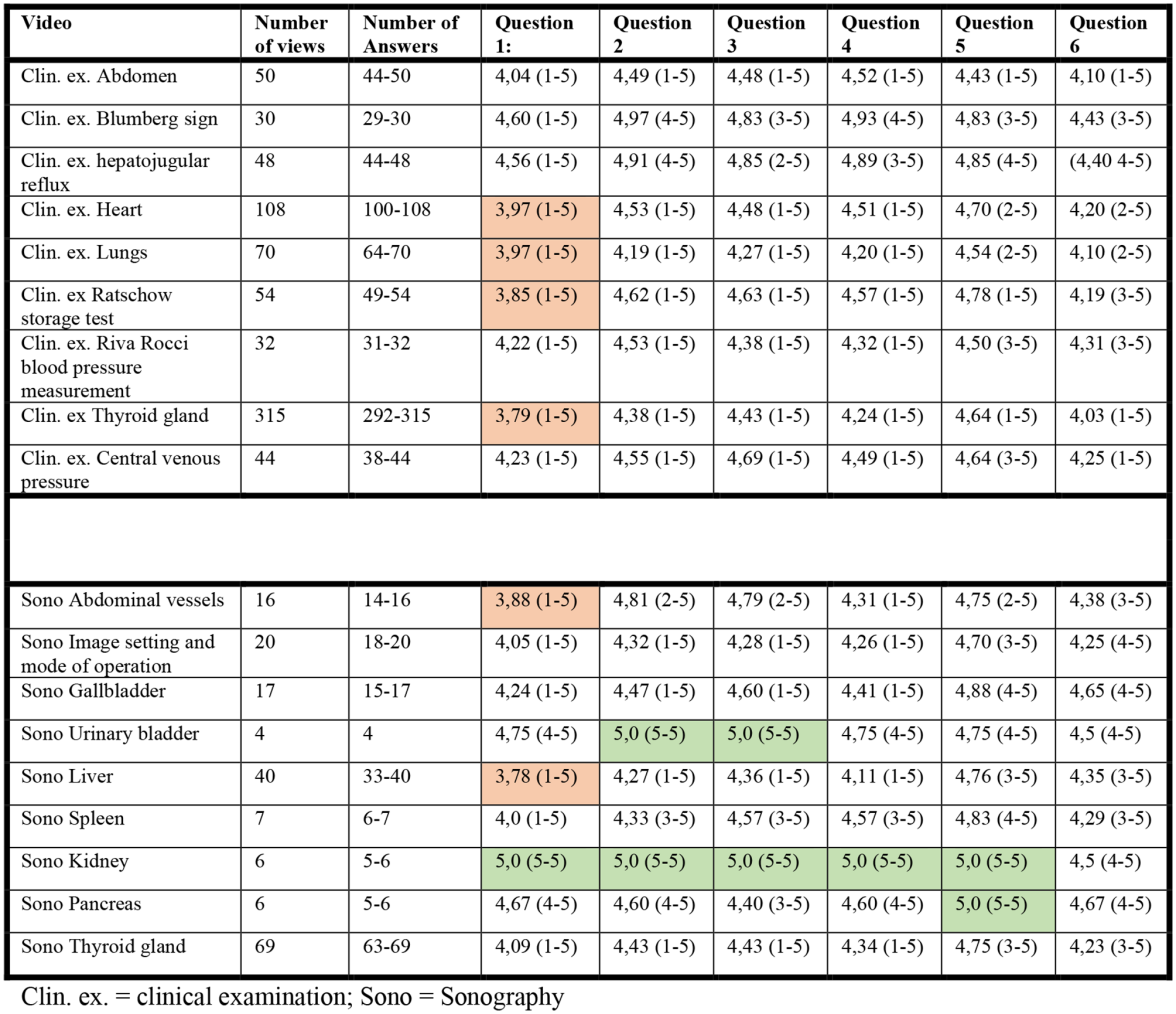
Presentation of the answers to the six questions relating to video quality, each given as a mean value with range. Answers with a mean value < 4 are marked red, those with a mean of 5 green

The specific evaluation of the responses per examination and question is shown in Supplementary material Tables 2–8 and presents the mean results of Table 4 in more detail. Only the video clinical examination of the abdomen was indicated as tending to be too long with a response rate > 10% by *n* = 8 (16%) of the users.

### Open questions

The first open-ended question was “What did you feel was missing in the video? What do you think could have been omitted?” This question was answered by 19 (2.0%) participants. In addition to many positive comments, constructive suggestions regarding the content of the videos were also provided. Missing examination steps or anatomical structures were specifically mentioned. Editorially, there was a request for the shortening or extension of certain sections of the video. The playback and speaking speed of the video was also criticized.

The second open-ended question was “Do you have any further comments? We look forward to your feedback!” and was answered by 121 (13%) participants. Here, 30 participants (23.80%) expressed satisfaction with the video.

59.5% (75 participants) had content-related improvement suggestions. These mainly included medical-anatomical suggestions for supplementing the videos. A more frequent representation of stages or exact orientation points were the most common topics. 16 participants (12.69%) found technical aspects worthy of improvement, where the speed or audio track along with different suggestions for speaker quality were most frequently noted.

## Discussion

The objective of this study was to investigate medical education videos for clinical examinations and sonography to gain a deeper insight / deeper understanding of what educational videos with what content are in demand by which target groups. Therefore, we focused on the video content and the user satisfaction.

The topic of instructional videos in medicine has been explored in various studies. For instance, Nason et al. [5] reported an improvement in training for male urinary catheterization through a related YouTube video, while Armstrong et al. [6] demonstrated enhanced psoriasis diagnostics via an instructional video on a dermatological platform. A 2021 study by Eccles [9] found that 93% of urology residents used YouTube as their primary source for educational videos, while only a minority were aware of the American Urological Association’s (AUA) quality-assured video platform. More recent surveys from 2023 confirmed this trend, with over 80% of residents continuing to rely on YouTube for surgical training videos [10]. The issue of a lack of quality-assured platforms had already been identified in data collected back in 2017 [11].

To fill this gap, the GeSRU Steps video platform was developed, offering supervised, standardized surgical videos produced the residents in urology. In a study by Nestler et al. these videos were generally well-received, technical quality was a common criticism, likely due to the absence of professional editing [12]. Given the overall positive feedback, the authors recommended that more resources be directed toward improving these educational tools.

In our study, the assessment of video quality based on questionnaire responses generally reflects a positive reception. Both categories of videos were rated highly for medical and technical quality, with average scores above 4 on a 5-point Likert scale for most questions, reflecting the more professional production of the videos by the AMBOSS partner. However, there were differences in specific aspects: sonography videos were rated higher for technical quality and clarity, likely due to the nature of sonographic procedures, which benefit from visual demonstrations. Conversely, clinical examination videos received slightly higher ratings for medical quality and practical application.

Feedback from open-ended responses provided further qualitative insights into the strengths and areas for improvement of the videos. Respondents valued the detailed and organized presentation of content, which enhanced their understanding of complex procedures. Suggestions for improvement included the addition of more detailed anatomical annotations and clearer explanations of certain techniques. These comments indicate a desire for even more detailed and accessible educational videos.

Overall, high scores were awarded in all questions (Table 4), and the questions about the time required to watch the videos (question 5) also received a particularly good response. In our view, this reflects the changed learning behavior of younger colleagues. Learning through traditional media such as books or atlases is increasingly perceived as time-consuming. A video is perceived here as the higher yield in the same unit of time.

The results of this study offer valuable insights into user preferences and perceptions of medical educational videos available on the AMBOSS platform. The data indicates a notable difference in viewership between clinical examination videos and sonography videos, with clinical examination content being significantly more popular. This could be due to the broader applicability and relevance of clinical examination skills in general medical practice, as opposed to the more specialized skill set of sonographies. The increased viewership of clinical examination videos, particularly among students in their final year of medical school, may be related to preparation for state exams, where clinical skills are more frequently assessed compared to sonography.

The demographic analysis of respondents shows that medical students, especially those nearing graduation, are the primary users of these videos. This trend highlights the importance of video content in practical training and the preparation of medical students for clinical practice. The preference for clinical examination videos among final-year students underscores their perceived value in mastering essential clinical skills prior to entering the professional field.

Additionally, the analysis revealed a correlation between respondents’ levels of training and their video preferences. Medical students and early-career doctors showed a preference for clinical examination videos, while more experienced practitioners and specialists favored sonography videos. This correlation suggests that educational content should be tailored to address the varying needs and expertise levels of its users.

This study also has limitations. For example, it should be noted that the results presented here evaluate the educational video project in general without any interventional design. It is not possible to draw conclusions about individual videos due to the wide range of responses (4-315 responses per video). Furthermore, only a random selection of educational videos was evaluated. The low response rate should also be mentioned, as the videos were only evaluated voluntarily, which can also result in a bias regarding particularly good or bad assessments in order to express the particular satisfaction or dissatisfaction with individual videos. Furthermore, the response rate should be put into perspective, as the number of questions answered was very good at 936 in total, but the total number of video uses was exorbitant at 1,643,274.

In view of the satisfaction surveyed, increasing funding should flow into the area of educational videos to further optimize the training of doctors in a timely manner.

## Conclusions

In conclusion, the study underscores the significant role of educational videos in medical training, particularly in enhancing practical skills for medical students and professionals. The findings suggest that both sonography and clinical examination videos are highly valued but serve different needs and preferences. To optimize the effectiveness of medical education, it is crucial to continuously refine video content based on user feedback and the evolving requirements of learners.

## Electronic supplementary material

Below is the link to the electronic supplementary material.

Supplementary Material 1

## Data Availability

Data is provided within the manuscript by supplementary information.

## Abbreviations

AUA: American Urological Association’s
GeSRU: German Society of Residents in Urology
