# Supplementary material for: Medical education videos – comparative analysis of sonography vs. clinical examination videos: user perception and educational value
Source: BMC Med Educ. 2024 Dec 18;24:1480. doi: 10.1186/s12909-024-06478-9 (PMC11653677; doi:10.1186/s12909-024-06478-9)
Supplement: Supplementary file 1 — Supplementary Material 1 [file 12909_2024_6478_MOESM1_ESM.docx]

**Supplementary material**

**Table 1:** The questionnaire translated from German into English. Questions 11-13 arose from the answer to question 10.

| **Number** | **Question** | **Answer Options** | | | | | |
| --- | --- | --- | --- | --- | --- | --- | --- |
|  |  | 1 | 2 | 3 | 4 | 5 | 0 |
| 1 | What is your first impression of the video, how satisfied are you with it? Just answer spontaneously from your gut. (1) means you are very dissatisfied with it. (5) means you are very satisfied. You can use the numbers in between to grade your opinion. | very dissatisfied | dissatisfied | partly partly | satisfied | very satisfied |  |
| 2 | Please indicate the extent to which you agree with the following statements. (1) means you do not agree at all. (5) means you agree completely. You can use the numbers in between to grade your opinion. The video presents medical facts correctly & understandably. | do not agree at all | do not agree | partly partly | agree | fully agree | I can't say |
| 3 | Please indicate the extent to which you agree with the following statements. (1) means you do not agree at all. (5) means you agree completely. You can use the numbers in between to grade your opinion. The video has a good technical quality (picture, sound etc.). | do not agree at all | do not agree | partly partly | agree | totaly agree | I can't say |
| 4 | Please indicate the extent to which you agree with the following statements. (1) means you do not agree at all. (5) means you agree completely. You can use the numbers in between to grade your opinion. The video helps me to grasp the learning content well. | do not agree at all | do not agree | partly partly | agree | totaly agree | I can't say |
| 5 | When you think about the learning content conveyed in the video: Do you think the time required to watch the video is appropriate? | inappropriate | rather inappropriate | partly partly | rather appropriate | appropriate | I can't say |
| 6 | How helpful did you find this video? | Generally not helpful: poor quality | Very limited help: poor quality, but contains little information | Partly helpful: moderate flow, some information included, but important information missing | Helpful: Good quality and good flow. Essential aspects considered | Extremely helpful: excellent quality / flow |  |
| 7 | You have indicated that you find the time spent (partly / rather) inappropriate in relation to the content conveyed. Do you think the video is too long or too short? | too short | too long |  |  |  |  |
| 8 | What was missing in the video? What do you think could have been left out? | open Question |  |  |  |  |  |
| 9 | Sex | female | male | diverse | Not specified |  |  |
| 10 | You are... | Student | MD in training | MD specialist |  |  |  |
| 11 | What semester are you in? | 1 | 2 | 3 | 4 | 5 | 6 |
| 12 | What year of your medical training are you in? | 1 | 2 | 3 | 4 | 5 | 6 |
| 13 | How many years have you been a medical specialist? | 1 | 2 | 3 | 4 | 5 | 6 |
| 14 | Do you have any other comments? We look forward to your feedback! | open Question |  |  |  |  |  |

**Table 2:** Question 1 - What is your first impression of the video, how satisfied are you with it? Just answer spontaneously from your gut. (1) means you are very dissatisfied with it. (5) means you are very satisfied. You can use the numbers in between to grade your opinion.

|  | **very dissatisfied** | **dissatisfied** | **partly partly** | **satisfied** | **very satisfied** | **total answers** |
| --- | --- | --- | --- | --- | --- | --- |
| Clin. ex. Abdomen | 2 | 5 | 5 | 15 | 23 | 50 |
| Clin. ex. Blumberg sign | 1 | 0 | 0 | 8 | 21 | 30 |
| Clin. ex. hepatojugular reflux | 2 | 2 | 0 | 7 | 37 | 48 |
| Clin. ex. Heart | 14 | 5 | 2 | 36 | 51 | 108 |
| Clin. ex. Lungs | 3 | 9 | 6 | 21 | 31 | 70 |
| Clin. ex Ratschow storage test | 9 | 4 | 1 | 12 | 28 | 54 |
| Clin. ex. Riva Rocci blood pressure measurement | 2 | 3 | 2 | 4 | 21 | 32 |
| Clin. ex Thyroid gland | 30 | 27 | 34 | 111 | 113 | 315 |
| Clin. ex. Central venous pressure | 3 | 3 | 1 | 11 | 26 | 44 |
| Sono Abdominal vessels | 3 | 1 | 0 | 3 | 9 | 16 |
| Sono Image setting and mode of operation | 3 | 1 | 0 | 4 | 12 | 20 |
| Sono Gallbladder | 2 | 1 | 0 | 2 | 12 | 17 |
| Sono Urinary bladder | 0 | 0 | 0 | 1 | 3 | 4 |
| Sono Liver | 8 | 1 | 1 | 12 | 18 | 40 |
| Sono Spleen | 1 | 0 | 0 | 3 | 3 | 7 |
| Sono Kidney | 0 | 0 | 0 | 0 | 6 | 6 |
| Sono Pancreas | 0 | 0 | 0 | 2 | 4 | 6 |
| Sono Thyroid gland | 7 | 3 | 5 | 16 | 38 | 69 |
| Total | 90 | 65 | 57 | 268 | 456 | 936 |

**Table 3:** Question 2 – Please indicate the extent to which you agree with the following statements. (1) means you do not agree at all. (5) means you agree completely. You can use the numbers in between to grade your opinion. The video presents medical facts correctly & understandably.

|  | **do not agree at all** | **do not agree** | **partly partly** | **agree** | **fully agree** | **I can't say** | **total answers** |
| --- | --- | --- | --- | --- | --- | --- | --- |
| Clin. ex. Abdomen | 2 | 1 | 1 | 11 | 32 | 3 | 50 |
| Clin. ex. Blumberg sign | 0 | 0 | 0 | 1 | 28 | 1 | 30 |
| Clin. ex. hepatojugular reflux | 0 | 0 | 0 | 4 | 40 | 4 | 48 |
| Clin. ex. Heart | 8 | 1 | 0 | 14 | 81 | 4 | 108 |
| Clin. ex. Lungs | 5 | 5 | 3 | 13 | 41 | 3 | 70 |
| Clin. ex Ratschow storage test | 3 | 0 | 2 | 4 | 43 | 2 | 54 |
| Clin. ex. Riva Rocci blood pressure measurement | 1 | 2 | 2 | 1 | 26 | 0 | 32 |
| Clin. ex Thyroid gland | 18 | 15 | 10 | 50 | 210 | 11 | 314 |
| Clin. ex. Central venous pressure | 2 | 0 | 1 | 7 | 28 | 6 | 44 |
| Sono Abdominal vessels | 0 | 1 | 0 | 0 | 15 | 0 | 16 |
| Sono Image setting and mode of operation | 2 | 1 | 0 | 2 | 14 | 1 | 20 |
| Sono Gallbladder | 1 | 1 | 0 | 1 | 12 | 2 | 17 |
| Sono Urinary bladder | 0 | 0 | 0 | 0 | 4 | 0 | 4 |
| Sono Liver | 3 | 2 | 0 | 6 | 22 | 7 | 40 |
| Sono Spleen | 0 | 0 | 1 | 2 | 3 | 1 | 7 |
| Sono Kidney | 0 | 0 | 0 | 0 | 5 | 0 | 5 |
| Sono Pancreas | 0 | 0 | 0 | 2 | 3 | 1 | 6 |
| Sono Thyroid gland | 4 | 3 | 3 | 7 | 50 | 2 | 69 |
| Total | 49 | 32 | 23 | 125 | 657 | 48 | 934 |

**Table 4:** Question 3 - Please indicate the extent to which you agree with the following statements. (1) means you do not agree at all. (5) means you agree completely. You can use the numbers in between to grade your opinion. The video has a good technical quality (picture, sound etc.).

|  | **do not agree at all** | **do not agree** | **partly partly** | **agree** | **totaly agree** | **I can't say** | **total answers** |
| --- | --- | --- | --- | --- | --- | --- | --- |
| Clin. ex. Abdomen | 2 | 1 | 2 | 9 | 32 | 3 | 49 |
| Clin. ex. Blumberg sign | 0 | 0 | 1 | 3 | 25 | 0 | 29 |
| Clin. ex. hepatojugular reflux | 0 | 1 | 0 | 4 | 41 | 1 | 47 |
| Clin. ex. Heart | 8 | 0 | 3 | 14 | 75 | 4 | 104 |
| Clin. ex. Lungs | 4 | 3 | 3 | 16 | 38 | 3 | 67 |
| Clin. ex Ratschow storage test | 3 | 0 | 1 | 4 | 41 | 1 | 50 |
| Clin. ex. Riva Rocci blood pressure measurement | 1 | 2 | 2 | 6 | 21 | 0 | 32 |
| Clin. ex Thyroid gland | 18 | 11 | 11 | 42 | 213 | 15 | 310 |
| Clin. ex. Central venous pressure | 2 | 0 | 0 | 9 | 32 | 1 | 44 |
| Sono Abdominal vessels | 0 | 1 | 0 | 0 | 13 | 0 | 14 |
| Sono Image setting and mode of operation | 1 | 2 | 1 | 1 | 13 | 2 | 20 |
| Sono Gallbladder | 1 | 0 | 1 | 0 | 13 | 0 | 15 |
| Sono Urinary bladder | 0 | 0 | 0 | 0 | 4 | 0 | 4 |
| Sono Liver | 5 | 0 | 0 | 3 | 28 | 4 | 40 |
| Sono Spleen | 0 | 0 | 1 | 1 | 5 | 0 | 7 |
| Sono Kidney | 0 | 0 | 0 | 0 | 6 | 0 | 6 |
| Sono Pancreas | 0 | 0 | 1 | 1 | 3 | 1 | 6 |
| Sono Thyroid gland | 6 | 1 | 2 | 5 | 49 | 4 | 67 |
| Total | 51 | 22 | 29 | 118 | 652 | 39 | 911 |

**Table 5:** Question 4 – Please indicate the extent to which you agree with the following statements. (1) means you do not agree at all. (5) means you agree completely. You can use the numbers in between to grade your opinion. The video helps me to grasp the learning content well.

|  | **do not agree at all** | **do not agree** | **partly partly** | **agree** | **totaly agree** | **I can't say** | **total answers** |
| --- | --- | --- | --- | --- | --- | --- | --- |
| Clin. ex. Abdomen | 1 | 1 | 3 | 8 | 31 | 3 | 47 |
| Clin. ex. Blumberg sign | 0 | 0 | 0 | 2 | 27 | 0 | 29 |
| Clin. ex. hepatojugular reflux | 0 | 0 | 1 | 3 | 42 | 1 | 47 |
| Clin. ex. Heart | 8 | 0 | 1 | 16 | 77 | 3 | 105 |
| Clin. ex. Lungs | 5 | 3 | 3 | 17 | 37 | 3 | 68 |
| Clin. ex Ratschow storage test | 3 | 1 | 2 | 4 | 43 | 1 | 54 |
| Clin. ex. Riva Rocci blood pressure measurement | 2 | 2 | 2 | 3 | 22 | 0 | 31 |
| Clin. ex Thyroid gland | 16 | 18 | 24 | 57 | 177 | 14 | 306 |
| Clin. ex. Central venous pressure | 3 | 1 | 1 | 5 | 33 | 1 | 44 |
| Sono Abdominal vessels | 1 | 1 | 1 | 2 | 11 | 0 | 16 |
| Sono Image setting and mode of operation | 1 | 2 | 0 | 4 | 12 | 1 | 20 |
| Sono Gallbladder | 1 | 1 | 1 | 1 | 13 | 0 | 17 |
| Sono Urinary bladder | 0 | 0 | 0 | 1 | 3 | 0 | 4 |
| Sono Liver | 4 | 1 | 4 | 5 | 22 | 4 | 40 |
| Sono Spleen | 0 | 0 | 1 | 1 | 5 | 0 | 7 |
| Sono Kidney | 0 | 0 | 0 | 0 | 6 | 0 | 6 |
| Sono Pancreas | 0 | 0 | 0 | 2 | 3 | 1 | 6 |
| Sono Thyroid gland | 5 | 1 | 5 | 9 | 44 | 4 | 68 |
| Total | 50 | 32 | 49 | 140 | 608 | 36 | 915 |

**Table 6:** Question 5 - When you think about the learning content conveyed in the video: Do you think the time required to watch the video is appropriate?

|  | **inappropriate** | **rather inappropriate** | **partly partly** | **rather appropriate** | **appropriate** | **I can't say** | **total answers** |
| --- | --- | --- | --- | --- | --- | --- | --- |
| Clin. ex. Abdomen | 1 | 0 | 8 | 8 | 32 | 1 | 50 |
| Clin. ex. Blumberg sign | 0 | 0 | 1 | 3 | 26 | 0 | 30 |
| Clin. ex. hepatojugular reflux | 0 | 0 | 0 | 7 | 41 | 0 | 48 |
| Clin. ex. Heart | 0 | 2 | 6 | 14 | 85 | 1 | 108 |
| Clin. ex. Lungs | 0 | 2 | 6 | 14 | 48 | 0 | 70 |
| Clin. ex Ratschow storage test | 1 | 0 | 1 | 6 | 46 | 0 | 54 |
| Clin. ex. Riva Rocci blood pressure measurement | 0 | 0 | 3 | 10 | 19 | 0 | 32 |
| Clin. ex Thyroid gland | 1 | 3 | 25 | 50 | 234 | 2 | 315 |
| Clin. ex. Central venous pressure | 0 | 0 | 3 | 10 | 31 | 0 | 44 |
| Sono Abdominal vessels | 0 | 1 | 0 | 1 | 14 | 0 | 16 |
| Sono Image setting and mode of operation | 0 | 0 | 1 | 4 | 15 | 0 | 20 |
| Sono Gallbladder | 0 | 0 | 0 | 2 | 15 | 0 | 17 |
| Sono Urinary bladder | 0 | 0 | 0 | 1 | 3 | 0 | 4 |
| Sono Liver | 0 | 0 | 2 | 5 | 30 | 3 | 40 |
| Sono Spleen | 0 | 0 | 0 | 1 | 5 | 1 | 7 |
| Sono Kidney | 0 | 0 | 0 | 0 | 6 | 0 | 6 |
| Sono Pancreas | 0 | 0 | 0 | 0 | 6 | 0 | 6 |
| Sono Thyroid gland | 0 | 0 | 2 | 13 | 54 | 0 | 69 |
| Total | 3 | 8 | 58 | 149 | 710 | 8 | 936 |

**Table 7:** Question 6 - How helpful did you find this video?

|  | Generally not helpful: poor quality | Very limited help: poor quality, but contains little information | Partly helpful: moderate flow, some information included, but important information missing | Helpful: Good quality and good flow. Essential aspects considered | Extremely helpful: excellent quality / flow | **total answers** |
| --- | --- | --- | --- | --- | --- | --- |
| Clin. ex. Abdomen | 1 | 0 | 2 | 37 | 10 | 50 |
| Clin. ex. Blumberg sign | 0 | 0 | 2 | 13 | 15 | 30 |
| Clin. ex. hepatojugular reflux | 0 | 0 | 0 | 29 | 19 | 48 |
| Clin. ex. Heart | 0 | 2 | 4 | 72 | 30 | 108 |
| Clin. ex. Lungs | 0 | 1 | 7 | 46 | 16 | 70 |
| Clin. ex Ratschow storage test | 0 | 0 | 2 | 40 | 12 | 54 |
| Clin. ex. Riva Rocci blood pressure measurement | 0 | 0 | 1 | 20 | 11 | 32 |
| Clin. ex Thyroid gland | 2 | 2 | 38 | 217 | 56 | 315 |
| Clin. ex. Central venous pressure | 1 | 0 | 2 | 25 | 16 | 44 |
| Sono Abdominal vessels | 0 | 0 | 1 | 8 | 7 | 16 |
| Sono Image setting and mode of operation | 0 | 0 | 0 | 15 | 5 | 20 |
| Sono Gallbladder | 0 | 0 | 0 | 6 | 11 | 17 |
| Sono Urinary bladder | 0 | 0 | 0 | 2 | 2 | 4 |
| Sono Liver | 0 | 0 | 4 | 18 | 18 | 40 |
| Sono Spleen | 0 | 0 | 1 | 3 | 3 | 7 |
| Sono Kidney | 0 | 0 | 0 | 3 | 3 | 6 |
| Sono Pancreas | 0 | 0 | 0 | 2 | 4 | 6 |
| Sono Thyroid gland | 0 | 0 | 6 | 41 | 22 | 69 |
| Total | 4 | 5 | 70 | 597 | 260 | 936 |

**Table 8:** Question 7 - You have indicated that you find the time spent (partly / rather) inappropriate in relation to the content conveyed. Do you think the video is too long or too short?

|  | too short | too long |
| --- | --- | --- |
| Clin. ex. Abdomen | 0 | 8 |
| Clin. ex. Blumberg sign | 0 | 1 |
| Clin. ex. hepatojugular reflux | 0 | 0 |
| Clin. ex. Heart | 2 | 7 |
| Clin. ex. Lungs | 1 | 7 |
| Clin. ex Ratschow storage test | 1 | 1 |
| Clin. ex. Riva Rocci blood pressure measurement | 1 | 2 |
| Clin. ex Thyroid gland | 10 | 19 |
| Clin. ex. Central venous pressure | 0 | 3 |
| Sono Abdominal vessels | 1 | 0 |
| Sono Image setting and mode of operation | 1 | 0 |
| Sono Gallbladder | 0 | 0 |
| Sono Urinary bladder | 0 | 0 |
| Sono Liver | 3 | 2 |
| Sono Spleen | 1 | 0 |
| Sono Kidney | 0 | 0 |
| Sono Pancreas | 0 | 0 |
| Sono Thyroid gland | 2 | 0 |
| Total | 23 | 50 |
